# Revisiting the eye opening response of the Glasgow Coma Scale

**DOI:** 10.4103/0972-5229.78231

**Published:** 2011

**Authors:** Taopheeq Bamidele Rabiu

**Affiliations:** Department of Neurological Surgery, University College Hospital, Ibadan, Nigeria

**Keywords:** Eye closure response, eye opening response, Glasgow Coma Scale, traumatic brain injury

## Abstract

The Glasgow Coma Scale (GCS), introduced by Teasdale and Jenneth in 1974, has received tremendous acclaim from clinicians and has been extensively used in clinical practice for the evaluation of the level of consciousness. The author notes that some traumatic brain injury patients close eyes in response to painful stimuli as opposed to the eye opening response to pain of the GCS. A revision of the eye opening response subsection of the GCS is suggested.

## Introduction

Since the presentation of the Glasgow Coma Scale (GCS) in 1974,[1] it has received tremendous acclaim from clinicians and has been extensively used in clinical practice for the evaluation of the level of consciousness in traumatic brain injury (TBI) patients. It has achieved widespread acceptance because of its highly accurate characterization of patients with depressed neurological function and its high level of inter-observer reliability.[1,2]

It is widely used to stratify head injury into mild (GCS 13–15), moderate (9–12) and severe (3–8) categories. It has also been used to compare the effectiveness of treatment options as well as prognosticate the outcome of TBI. In addition, it has been incorporated into many trauma and critical illness classification systems such as the trauma score, revised trauma score,[3] and the Acute Physiology and Chronic Health Evaluation (APACHE) II score.

The scale is, however, not without its shortcomings. A result of its perceived limited nature is the design of several alternative scales which are aimed at addressing its weaknesses and provide clinicians with tools which may be useful when the GCS is adjudged inadequate.

## Limitations

Despite the almost universal acceptance of the GCS, several shortcomings of the scale have been identified. These include the inability to assess eye opening in patients with periorbital trauma, the loss of verbal response in intubated patients, as well as the non-inclusion of brain stem reflexes.[4] In particular, the scale does not include the pupillary examination, a standard and integral part of the evaluation of neurological patients.[5,6]

In a comprehensive review, Sternbach[7] detailed the strengths of the scale as well as the observed weaknesses. He evaluated the various scales developed to overcome the perceived deficiencies of the GCS, including the Reaction Level Scale (RLS85), the Maryland Coma Scale, the Innsbruck Coma Scale and the Glasgow-Liege Scale. Although the various competing scales have found some acceptance in certain places, the author concluded, “the GCS remains the most universally utilized level of consciousness scale worldwide…, the GCS, by virtue of its simplicity, seems destined to be used in emergency medicine for some time.”

## New Observations

In describing the eye opening response, Teasdale and Jenneth stated, “Spontaneous eye opening … indicates that the arousal mechanisms in the brainstem are active”.[1] Thus, the upward progression of a patient along the scale from a state of no eye opening to eye opening to pain, eye opening to speech and spontaneous eye opening indicates clinical improvement and recovery from impaired consciousness.

In my practice, I have observed a subset of TBI patients who strongly close their eyes in response to painful stimuli. This is usually an unexpected response as such patients localize pain, make incomprehensible sounds and would otherwise be expected to open eyes in response to pain. At present, no specific factor which could account for this response and which distinguishes the patients from their counterparts who open eyes to pain has been identified.

A prospective observational study to define the clinical characteristics of this subset of patients and to compare them with their counterparts who open eyes to painful stimuli, with a view to determining the role of the eye opening response in the overall outcome following TBI, is desirable. Multicenter observations are needed to validate this deviation from traditional concepts. A revision of the GCS may result from such collaborative effort.

## Conclusion

A subset of TBI patients exhibits non-conventional eye closure response to pain and does not fit into the GCS as it presently stands. Does this “eye closure response to pain” represent a possible equivalent of eye opening response to pain and can it be included in a future modification of the assessment scale? Do other clinicians observe similar phenomenon?
